# Supplementary material for: Splice-Junction-Based Mapping of Alternative Isoforms in the Human Proteome
Source: Cell Rep. Author manuscript; Available in PMC 2020 Jan 15. (PMC6961840; doi:10.1016/j.celrep.2019.11.026)
Supplement: 2 [file NIHMS1546469-supplement-2.zip › DF1/PXD006675/Aorta/Aorta_9_ELN_AQLLPGALAAAK.pdf]

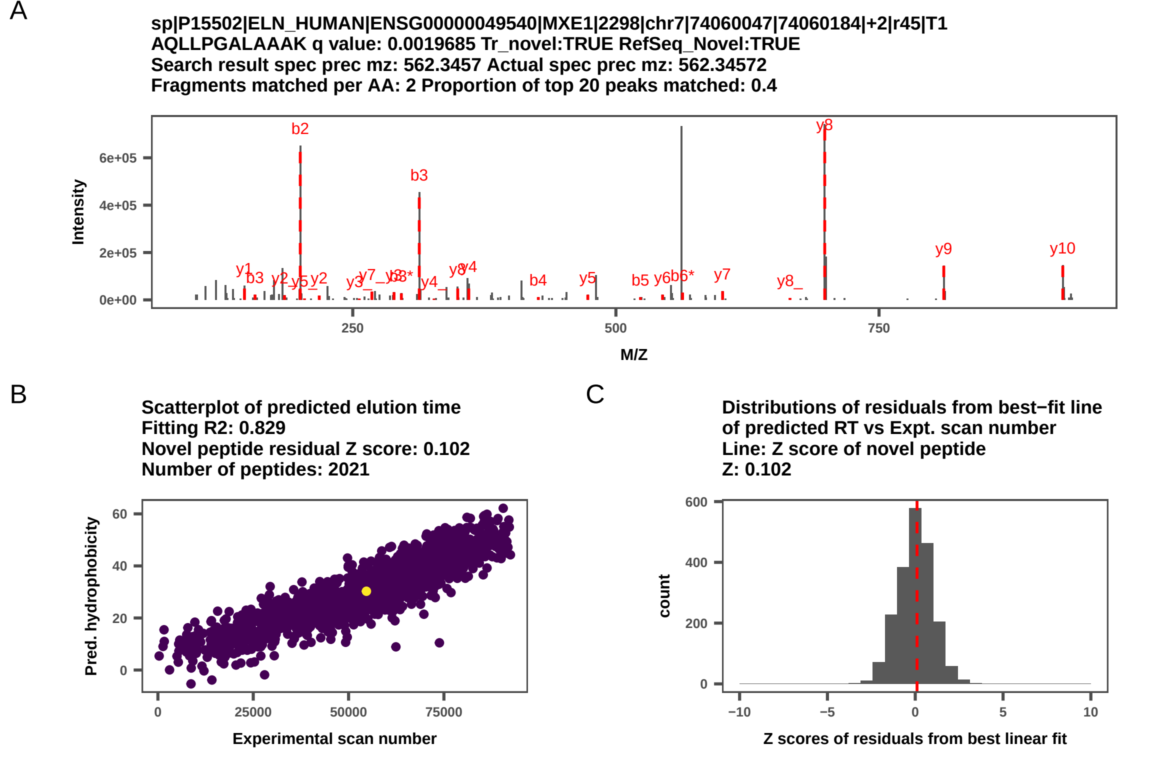
A
sp|P15502|ELN_HUMAN|ENSG00000049540|MXE1|2298|chr7|74060047|74060184|+2|r45|T1
AQLLPGALAAAK q value: 0.0019685 Tr_novel:TRUE RefSeq_Novel:TRUE
Search result spec prec mz: 562.3457 Actual spec prec mz: 562.34572
Fragments matched per AA: 2 Proportion of top 20 peaks matched: 0.4
Intensity
6e+05
4e+05
2e+05
0e+00
b2
b3
y8
y9
y10
y1
b3
y2
y5_
y2
y3_
y7_
y3
b3*
y4_
y8
y4
b4
y5
b5
y6
b6*
y7
y8_
250
500
750
M/Z
B
Scatterplot of predicted elution time
Fitting R2: 0.829
Novel peptide residual Z score: 0.102
Number of peptides: 2021
Pred. hydrophobicity
60
40
20
0
0
25000
50000
75000
Experimental scan number
C
Distributions of residuals from best−fit line
of predicted RT vs Expt. scan number
Line: Z score of novel peptide
Z: 0.102
count
600
400
200
0
−10
−5
0
5
10
Z scores of residuals from best linear fit
